# Knowledge, Attitudes, and Practices Toward Telemedicine Among Physicians in the United Arab Emirates: A Cross-Sectional Study

**DOI:** 10.7759/cureus.107199

**Published:** 2026-04-16

**Authors:** Rashida Dhilawala, Farah A Abu Assi, Ahd Elmahi, Amna Ahmed Al Ali, Sama Abushairah, Wafa K Alnakhi, Ali Shorbagi, Amal Hussein

**Affiliations:** 1 College of Medicine, University of Sharjah, Sharjah, ARE; 2 Department of Family and Community Medicine and Behavioral Sciences/Public Health, University of Sharjah, Sharjah, ARE; 3 Department of Gastroenterology, University Hospital Sharjah, Sharjah, ARE

**Keywords:** attitude, digital health communications, knowledge, practice, practicing physicians, telemedicine, united arab emirates (uae)

## Abstract

Background

Telemedicine has emerged as a pivotal tool to address growing healthcare demands and improve access to care, particularly following the COVID-19 pandemic. While the United Arab Emirates (UAE) has made substantial investments in digital health infrastructure, variations in physicians’ readiness and acceptance remain. This study aimed to assess physicians’ knowledge, attitudes, and practices (KAP) regarding telemedicine and to identify demographic and professional factors associated with these domains.

Methods

A descriptive cross-sectional study was conducted among practicing physicians in public and private healthcare facilities across the UAE between March and September 2023. A nonprobability convenience sampling method, supplemented by snowball sampling, was used to recruit participants through a structured, self-administered bilingual questionnaire developed for this study, which assessed demographics and KAP toward telemedicine. This approach was adopted due to challenges in accessing physicians across diverse clinical settings and to ensure an adequate sample size. A total of 309 complete responses were analyzed. KAP scores were categorized into low, moderate, and high levels. Descriptive statistics, chi-square tests, and multivariable ordinal logistic regression were performed using IBM SPSS Statistics for Windows, version 22.0 (released 2013; IBM Corp., Armonk, NY, USA), with significance set at p < 0.05.

Results

Among participants, 51.4% demonstrated high knowledge, 59.2% had positive attitudes, and 63.2% reported good telemedicine practices. Knowledge was significantly associated with years of experience, emirate of practice, and healthcare sector. Physicians in private facilities had higher odds of good knowledge (adjusted OR (AOR) = 2.31, p < 0.001), while those in obstetrics and gynecology had lower odds compared with surgeons (AOR = 0.28, p = 0.016). Positive attitudes were more likely among physicians in medicine and obstetrics/gynecology and among those practicing in Dubai. Good practice was significantly associated with family medicine specialty and geographic location, with regional variation across emirates.

Discussion

Overall, UAE physicians exhibited favorable engagement with telemedicine; however, knowledge and adoption levels were lower than those reported in many Western countries and varied across specialties and regions. Disparities between the public and private sectors and among emirates suggest unequal access to training and infrastructure. These findings align with regional literature indicating moderate but growing acceptance, underscoring the need for targeted educational and institutional support.

Conclusions

Telemedicine is widely accepted among physicians in the UAE, with generally high levels of knowledge, positive attitudes, and good practice. Nonetheless, specialty-specific gaps, public-sector limitations, and geographic disparities persist. These findings highlight the need for targeted, specialty-specific training programs and capacity-building workshops, particularly within public healthcare settings, to address identified gaps. Additionally, standardized national guidelines and enhanced system-level support are essential to optimize telemedicine integration and ensure equitable, high-quality digital healthcare delivery nationwide.

## Introduction

Facing the escalating demands and limited accessibility that challenge healthcare systems globally, telemedicine has become an essential strategy for delivering prompt and effective medical care. The Institute of Medicine defines telemedicine as “the use of electronic information and communications technologies to provide and support health care when distance separates the participants” [1]. Telemedicine comprises several key pillars that together enable comprehensive remote healthcare delivery. Telediagnosis involves the remote identification and evaluation of diseases through digital imaging and sensor data [2], while teleconsultation allows real-time or asynchronous interactions between patients and healthcare professionals for clinical evaluation and treatment planning [3]. Telemonitoring supports continuous or periodic remote tracking of patients’ health status, which is especially useful for chronic disease management [4]. Telepharmacy delivers pharmaceutical services remotely, including prescription management and medication counseling [5], and additionally, teletherapy and telepsychiatry provide remote therapeutic and mental health interventions, enhancing mental healthcare accessibility, particularly in underserved areas [6]. Together, these pillars form the foundation of telemedicine’s capacity to improve access, efficiency, and patient outcomes across diverse healthcare contexts [2].

Telemedicine has undergone a marked surge globally, especially in response to the recent COVID-19 pandemic. Healthcare systems worldwide have adopted this technology to maintain the continuity of care amid widespread lockdowns and social distancing mandates. Countries with well-established digital infrastructure, like the United States, Canada, and several EU nations, were the first to integrate teleconsultation platforms into primary and specialized care services. However, technological limitations in addition to inadequate policy frameworks and institutional capabilities posed significant impediments to effective implementation, particularly in least developed countries [7].

In the United Arab Emirates (UAE), investment in digital infrastructure began as early as 2013 in government entities, following a well-structured plan to utilize telemedicine as a viable, long-term model of care. The COVID-19 pandemic played a major role in accelerating the adoption of telemedicine in all Emirates. In the UAE, telemedicine adoption has been supported by multiple platforms. Emirates Health Services currently uses the “Metaverse” platform to provide teleconsultation and treatment planning. The Dubai Health Authority (DHA) launched “Doctor for Every Citizen” in December 2019, offering free initial and follow-up consultations with DHA-certified physicians. Meanwhile, in Abu Dhabi, the Department of Health introduced the “RemoteCare” app, a telemonitoring tool that delivers healthcare to patients in their homes. Research conducted by Abdool et al. (2021) [8] highlights the UAE’s rapid adoption and user acceptance of telemedicine, with most participants expressing positive attitudes, especially for follow-ups and nonurgent care. Nevertheless, issues such as concerns about data privacy, perceived limitations in clinical accuracy, and insufficient training were also prominent, suggesting areas for targeted policy and system-level improvements [3].

The primary objective of this study was to evaluate physicians’ knowledge, attitudes, and practices (KAP) toward telemedicine in the UAE. The secondary objective was to examine the association between KAP outcomes and physician characteristics, including age, gender, specialty, years of experience, healthcare sector, and emirate of practice.

## Materials and methods

Study design

This study employed a descriptive cross-sectional design. A structured, self-administered questionnaire was used to gather data from participants across various healthcare settings, including both public and private institutions (Appendix A). Participants were recruited using a nonprobability sampling approach, primarily convenience sampling, supplemented by snowball recruitment, whereby initial respondents were encouraged to share the survey with eligible colleagues.

Inclusion and exclusion criteria

Inclusion criteria included all Arabic- and/or English-speaking physicians actively practicing within public and private healthcare facilities in the UAE during the data collection period (March 6 to September 15, 2023), irrespective of medical specialty. Physicians not engaged in clinical practice, along with those in training, including medical interns, were excluded. Additionally, questionnaires with incomplete responses or missing critical data were omitted from the final analysis to preserve data quality and integrity.

Sample size

The expected proportion was derived from Datta et al.’s study, which reported a prevalence of telemedicine-related knowledge of 74.09% [9]. Assuming a similar prevalence in our target population and a margin of error of 5%, the sample size was calculated using the following formula:

$n = \frac{4p(1 - p)}{SE^2},$

where n represents the required sample size, p is the expected proportion (prevalence) expressed as a decimal, and SE is the acceptable margin of error [10]. Substituting the values,

$n = 1540 \times 0.7409 \times (1 - 0.7409) = 295.6,$

which was rounded to 296 participants. Accordingly, the minimum required sample size was determined to be 296 participants. A total of 309 valid responses were ultimately collected and included in the final analysis, exceeding the minimum requirement.

Data collection

Data were collected using a structured, self-administered questionnaire developed de novo by the research team following an extensive review of the relevant literature. Prior to questionnaire development, published studies on telemedicine adoption and KAP among healthcare professionals were reviewed to determine whether a standardized or validated instrument assessing physicians’ telemedicine-related KAP was available. However, no validated tool specifically addressing physicians’ KAP regarding telemedicine was identified. Therefore, all questionnaire items were independently constructed by the authors based on the objectives of the study. The questionnaire did not reproduce or adapt any previously copyrighted or proprietary instruments; therefore, no licensing permissions were required. The initial version was prepared in English and reviewed by two independent faculty members to ensure content validity. Subsequently, the questionnaire was translated into Arabic and underwent a second round of expert review, with modifications incorporated based on their feedback to enhance clarity and appropriateness.

KAP scores were calculated according to a predefined scoring framework developed by the authors. Knowledge was assessed using four scored items, with each correct response assigned a score of 1 and incorrect responses scored as 0, resulting in a total possible knowledge score ranging from 0 to 4. Based on the total score obtained, participants were categorized as having low (0-1), moderate (2), or high (3-4) knowledge levels. Attitude was assessed using items with a maximum attainable score of 15. Total attitude scores were categorized as negative (0-5), neutral (6-10), or positive (11-15) attitudes toward telemedicine. Practice was evaluated using items with a maximum possible score of 10. Total practice scores were classified as poor (0-4), moderate (5-7), or good (8-10) practice levels. These categorizations were defined a priori by the research team to facilitate standardized interpretation of KAP levels among participants.

To maximize accessibility and response rates, the questionnaire was disseminated electronically via social media platforms, email, and online survey platforms. A pilot study was conducted among a subset of the target population to assess the tool’s clarity, validity, and reliability prior to full-scale deployment.

Study variables

Demographic and professional characteristics, including age, gender, medical specialty, years of clinical experience, emirate of practice, and type of healthcare facility, were collected to examine their potential associations with the study outcomes. The primary outcome variables were physicians’ KAP related to telemedicine in the UAE.

Physicians’ knowledge of telemedicine was measured through four closed-ended questions assessing awareness of telemedicine concepts, operational guidelines, and technical aspects, with responses captured using a Likert scale [11]. Attitudes were evaluated with seven Likert-scale items measuring perceptions, acceptance, and opinions regarding the benefits, limitations, and ethical considerations of telemedicine in clinical care [11]. Practices were assessed using ten items focusing on real-life application, including frequency of use, types of services provided, and perceived ease or barriers, with a combination of Likert and linear rating scales employed [11,12].

The final questionnaire consisted of 33 items divided into four sections: demographics, knowledge, practice, and attitude. To ensure response validity and prevent duplication, participants were asked to enter partial, nonidentifiable information (the last three digits of their mobile number and the first three letters of their surname).

Statistical analysis

All data were coded, entered, and analyzed using IBM SPSS Statistics for Windows, version 22.0 (released 2013; IBM Corp., Armonk, NY, USA). Descriptive statistics were used to summarize participant demographics and responses related to KAP. Results were presented as frequencies and percentages for categorical variables.

Scores were computed according to a predefined framework developed by the authors based on commonly used approaches in KAP research [13]. Knowledge items were scored (1 for positive responses and 0 for negative), yielding total scores ranging from 0 to 4 and categorized as low (0-1), moderate (2), or high (3-4). Attitude scores ranged from 0 to 15, with groups of negative (0-5), neutral (6-10), and positive (11-15). Lastly, practice scores ranged from 0 to 10, classified as poor (0-4), moderate (5-7), and good (8-10). Scores were categorized according to predefined thresholds developed by the authors, and no proprietary or licensed instruments were used. This scoring approach permitted standardized quantification of KAP levels across participants.

Prior to conducting the primary comparative analysis, the construct validity of the newly developed KAP questionnaire was evaluated using the full study sample. Construct validity was assessed through exploratory factor analysis (EFA) with principal component analysis and varimax rotation. The Kaiser-Meyer-Olkin (KMO) measure and Bartlett’s test of sphericity indicated that the data were suitable for factor analysis (KMO = 0.743; χ² = 1020.31, p < 0.001). The final EFA yielded a three-factor solution, with factors representing KAP, each with factor loadings greater than 0.40. Reliability was assessed using Cronbach’s α coefficient, which demonstrated acceptable internal consistency for the individual factors (knowledge: α = 0.81, attitude: α = 0.791, practice: α = 0.76). No pilot testing was conducted, but construct validity and reliability were assessed prior to the final analysis. These results indicate that the KAP questionnaire is both valid and reliable for use in telemedicine-related research.

Associations between KAP categories and demographic or professional characteristics (including specialty, years of experience, emirate of practice, and type of healthcare facility) were conducted using the chi-square test. To further identify independent predictors of higher KAP levels, ordinal regression models were employed, with results expressed as adjusted ORs (aORs) and corresponding 95% CIs. aORs were calculated using

$\mathrm{OR} = e^{\beta},$

where β represents the regression coefficient, and the 95% CI was calculated via

$\mathrm{CI}_{95\%} = \left( e^{(\beta - 1.96 \times SE(\beta))}, \; e^{(\beta + 1.96 \times SE(\beta))} \right)$

The proportional odds assumption was assessed using the test of parallel lines and was not violated (p > 0.05). A p-value of <0.05 was considered statistically significant. Incomplete and duplicate responses were excluded from all analyses to preserve data quality and integrity.

## Results

Table 1 presents the demographic characteristics of the participating physicians. Most respondents were in the 35-50 years age group (50.2%), followed by those younger than 35 years (26.2%) and a smaller proportion older than 50 years (23.6%). In terms of gender, the distribution was fairly balanced, with a slight predominance of female physicians (53.7%) compared with males (46.3%).

**Table 1:** Sociodemographic and professional characteristics of the participants (N = 309)

Variable	Category	n (%)
Age group	Less than 35 years	81 (26.2)
	35-50 years	155 (50.2)
	More than 50 years	73 (23.6)
Gender	Male	143 (46.3)
	Female	166 (53.7)
Specialty	Medicine	133 (43.0)
	Surgery	89 (28.8)
	Pediatrics	46 (14.9)
	Obstetrics/gynecology	22 (7.1)
	Family medicine	19 (6.1)
Years of experience	Less than 10 years	107 (34.6)
	10 or more years	202 (65.4)
Working Emirate	Dubai	117 (37.9)
	Sharjah	119 (38.5)
	Abu Dhabi	56 (18.1)
	Other	17 (5.5)
Healthcare facility	Public	166 (53.7)
	Private	143 (46.3)

When looking at specialty, the largest group was from medicine (43.0%), followed by surgery (28.8%). Pediatrics (14.9%), obstetrics/gynecology (7.1%), and family medicine (6.1%) accounted for smaller proportions. With respect to professional experience, nearly two-thirds (65.4%) had 10 or more years of experience, while about one-third (34.6%) reported fewer than 10 years. Geographical distribution showed that participants were fairly evenly distributed between Sharjah (38.5%) and Dubai (37.9%), with fewer practicing in Abu Dhabi (18.1%) and other emirates (5.5%). Finally, regarding healthcare facilities, a slightly higher proportion worked in public institutions (53.7%) compared with private ones (46.3%).

The distribution of knowledge levels regarding telemedicine showed that just over half of participants had a high level of knowledge (51.4%), while 36.9% were in the moderate category, and 11.7% demonstrated low knowledge. Attitudes toward telemedicine were largely positive, with 59.2% expressing a positive outlook, 33.7% neutral, and 7.1% negative. In terms of practice, most participants exhibited good practice (63.2%), followed by moderate practice (30.7%), while a minority demonstrated poor practice (6.1%). Overall, the findings indicate that most physicians had strong knowledge, favorable attitudes, and good practices toward telemedicine, with a smaller subgroup remaining at moderate or low levels.

Table 2 presents the relationship between the participants’ knowledge regarding telemedicine and their demographic and professional characteristics. Gender was not a statistically significant factor in determining knowledge level (p = 0.267), as almost equal proportions of high knowledge were found among male (48.3%) and female (54.2%) participants. Similarly, specialty did not reveal any significant association with knowledge levels (p = 0.361).

**Table 2:** Chi-square test of association between knowledge scores and demographic characteristics (N = 309) Items in this table were assessed using a Likert-type scale [11]. Scores were categorized according to the study’s KAP framework [13]. KAP, knowledge, attitudes, and practices

Characteristic	High (n)	Moderate (n)	Low (n)
Age group (0.017)			
Less than 35 years	32	42	7
35-50 years	88	50	17
More than 50 years	39	22	12
Gender (0.267)			
Female	90	61	15
Male	69	53	21
Specialty (0.361)			
Medicine	68	54	11
Surgery	43	30	16
Pediatrics	22	19	5
Obstetrics/gynecology	16	4	2
Family medicine	10	7	2
Years of experience (0.050)			
Less than 10 years	49	49	9
10 or more years	110	65	27
Working Emirate (0.012)			
Dubai	71	37	9
Sharjah	45	57	17
Abu Dhabi	32	16	8
Other	11	4	2
Healthcare facility (0.012)			
Public	97	56	13
Private	62	58	23

Years of professional experience had a borderline significant association with knowledge (p = 0.050). Doctors who have been practicing for 10 years or more were more likely to have high knowledge (54.5%) than those with less than 10 years of experience (45.8%). Other factors such as age, emirate of work, and healthcare facility type were not associated with participants’ knowledge levels.

Table 3 demonstrates the association between participants’ attitudes toward telemedicine and demographic and professional characteristics. Chi-square analysis showed that none of the examined variables, including age, gender, specialty, years of experience, working emirate, and type of healthcare facility, were significantly associated with attitude levels (all p > 0.05). Therefore, no meaningful relationship was observed between participants’ attitudes toward telemedicine and any of the independent variables assessed.

**Table 3:** Chi-square test of association between attitude scores and demographic characteristics (N = 309) Items in this table were assessed using a Likert-type scale [11]. Scores were categorized according to the study’s KAP framework [13]. KAP, knowledge, attitudes, and practices

Characteristic	Negative (n)	Neutral (n)	Positive (n)
Age group (0.567)			
Less than 35 years	3	26	52
35-50 years	14	51	90
More than 50 years	5	27	41
Gender (0.100)			
Female	13	47	106
Male	9	57	77
Specialty (0.337)			
Medicine	7	39	87
Surgery	9	38	42
Pediatrics	3	17	26
Obstetrics/gynecology	2	4	16
Family medicine	1	6	12
Years of experience (0.477)			
Less than 10 years	5	37	65
10 or more years	17	67	118
Working Emirate (0.129)			
Dubai	4	35	78
Sharjah	11	39	69
Abu Dhabi	6	25	25
Other	1	5	11
Healthcare facility (0.245)			
Public	12	49	105
Private	10	55	78

Table 4 presents the association between participants’ practice levels of telemedicine and demographic and professional characteristics. A statistically significant association was observed only for the emirate of work (p = 0.002). Participants working in Dubai demonstrated the highest proportion of good practice (76.1%), followed by those in Abu Dhabi (60.7%) and Sharjah (53.8%). No significant associations were found between practice level and age, gender, specialty, years of experience, or type of healthcare facility (all p > 0.05).

**Table 4:** Chi-square test of association between practice scores and demographic characteristics (N = 309) Items in this table were assessed using a Likert-type and linear scale [11,12]. Scores were categorized according to the study’s KAP framework [13]. KAP, knowledge, attitudes, and practices

Characteristic	Good (n)	Moderate (n)	Poor (n)
Age group (0.671)			
Less than 35 years	56	21	4
35-50 years	96	48	11
More than 50 years	43	26	4
Gender (0.305)			
Female	106	53	7
Male	89	42	12
Specialty (0.176)			
Medicine	88	38	7
Surgery	58	24	7
Pediatrics	23	18	5
Obstetrics/gynecology	17	5	0
Family medicine	9	10	0
Years of experience (0.685)			
Less than 10 years	70	32	5
10 or more years	125	63	14
Working Emirate (0.002)			
Dubai	89	24	4
Sharjah	64	42	13
Abu Dhabi	34	20	2
Other	8	9	0
Healthcare facility (0.267)			
Public	101	56	9
Private	94	39	10

Table 5 presents the multivariable logistic regression analysis of factors associated with physicians’ telemedicine knowledge levels. Specialty, geographical location, and type of healthcare facility were significant predictors. Compared with surgeons, physicians in obstetrics and gynecology had significantly lower odds of good knowledge (AOR = 0.280; 95% CI: 0.099-0.790; p = 0.016). Physicians working in Dubai (AOR = 0.344; 95% CI: 0.200-0.590; p < 0.001) and Abu Dhabi (AOR = 0.489; 95% CI: 0.255-0.936; p = 0.031) also had lower odds of good knowledge compared with those in Sharjah. In contrast, physicians employed in private healthcare facilities had higher odds of good knowledge than those in public institutions (AOR = 2.312; 95% CI: 1.452-3.684; p < 0.001).

**Table 5:** Multivariable ordinal logistic regression analysis of knowledge scores with demographic variables AOR, adjusted OR

Characteristic	AOR (95% CI)	p-Value
Age group		
More than 50 years	1.00	-
35-50 years	1.050 (0.582-1.896)	0.871
Less than 35 years	2.638 (0.945-7.367)	0.05
Gender		
Male	1.00	-
Female	0.851 (0.527-1.374)	0.509
Specialty		
Surgery	1.00	-
Medicine	0.781 (0.453-1.344)	0.373
Pediatrics	0.825 (0.408-1.670)	0.594
Obstetrics/gynecology	0.280 (0.099-0.790)	0.016
Family medicine	0.829 (0.301-2.282)	0.716
Years of experience		
Less than 10 years	1.00	-
10 or more years	1.543 (0.659-3.615)	0.317
Working Emirate		
Sharjah	1.00	-
Dubai	0.344 (0.200-0.590)	<0.001
Abu Dhabi	0.489 (0.255-0.936)	0.031
Other	0.397 (0.137-1.148)	0.088
Healthcare facility		
Public	1.00	-
Private	2.312 (1.452-3.684)	<0.001

Table 6 presents the multivariable logistic regression analysis of factors associated with physicians’ attitudes toward telemedicine. Specialty and geographical location were significant predictors. Compared with surgeons, physicians in medicine (AOR = 2.237; 95% CI: 1.271-3.935; p = 0.005) and obstetrics and gynecology (AOR = 2.821; 95% CI: 1.003-7.933; p = 0.049) had higher odds of positive attitudes. Additionally, physicians working in Dubai had higher odds of positive attitudes compared with those in Sharjah (AOR = 1.791; 95% CI: 1.025-3.130; p = 0.04).

**Table 6:** Multivariable ordinal logistic regression analysis of attitude scores with demographic variables AOR, adjusted OR

Characteristic	AOR (95% CI)	p-Value
Age group		
More than 50 years	1.00	-
35-50 years	0.868 (0.475-1.584)	0.644
Less than 35 years	1.594 (0.570-4.459)	0.374
Gender		
Male	1.00	-
Female	1.048 (0.640-1.716)	0.852
Specialty		
Surgery	1.00	-
Medicine	2.237 (1.271-3.935)	0.005
Pediatrics	1.637 (0.798-3.357)	0.179
Obstetrics/gynecology	2.821 (1.003-7.933)	0.049
Family medicine	1.853 (0.650-5.280)	0.248
Years of experience		
Less than 10 years	1.00	-
10 or more years	1.162 (0.506-2.670)	0.724
Working Emirate		
Sharjah	1.00	-
Dubai	1.791 (1.025-3.130)	0.04
Abu Dhabi	0.590 (0.309-1.126)	0.110
Other	1.105 (0.375-3.254)	0.856
Healthcare facility		
Public	1.00	-
Private	0.640 (0.398-1.029)	0.056

Table 7 presents the multivariable ordinal logistic regression analysis of factors associated with physicians’ telemedicine practices. Specialty and geographical location were significant predictors. Compared with surgeons, physicians in family medicine had higher odds of higher levels of telemedicine practice (AOR = 3.121; 95% CI: 1.112-8.767; p = 0.031). In terms of regional variation, physicians working in Dubai had lower odds of higher levels of telemedicine practice compared with those in Sharjah (AOR = 0.350; 95% CI: 0.193-0.631; p < 0.001).

**Table 7:** Multivariable ordinal logistic regression analysis of practice scores with demographic variables AOR, adjusted OR

Characteristic	AOR (95% CI)	p-Value
Age group		
More than 50 years	1.00	-
35-50 years	0.891 (0.478-1.662)	0.717
Less than 35 years	0.527 (0.186-1.496)	0.229
Gender		
Male	1.00	-
Female	1.302 (0.774-2.188)	0.320
Specialty		
Surgery	1.00	-
Medicine	0.794 (0.436-1.445)	0.450
Pediatrics	1.790 (0.799-3.518)	0.172
Obstetrics/gynecology	0.557 (0.183-1.692)	0.302
Family medicine	3.121 (1.112-8.767)	0.031
Years of experience		
Less than 10 years	1.00	-
10 or more years	0.916 (0.394-2.132)	0.839
Working Emirate		
Sharjah	1.00	-
Dubai	0.350 (0.193-0.631)	<0.001
Abu Dhabi	0.856 (0.439-1.672)	0.650
Other	1.870 (0.670-5.223)	0.232
Healthcare facility		
Public	1.00	-
Private	0.852 (0.522-1.394)	0.524

## Discussion

The COVID-19 pandemic accelerated telemedicine use worldwide. In the United States, physician telehealth visits increased from 15.4% in 2019 to 86.5% in 2021, with three-quarters of primary care doctors reporting comparable quality of care to in-person visits [14]. In our UAE study, 51.4% of physicians demonstrated high knowledge, 59.2% held positive attitudes, and 63.2% reported good practice. These findings indicate that while most physicians embrace telemedicine, ratios remain below those reported in Western settings.

Naqvi et al. reported a higher level of knowledge, with 73.7% of healthcare workers in the UAE demonstrating familiarity with telemedicine [15]. Regional comparisons show mixed results. A Saudi hospital study found that 63.7% of staff had limited knowledge, with only 11% reporting good knowledge [16], whereas an Egyptian research study reported that two-thirds of physicians were knowledgeable [15]. UAE physicians fall between some MENA countries and the higher global averages, suggesting room for educational initiatives to raise knowledge levels.

In addition, Naqvi et al. found that nearly 60% of UAE physicians reported positive attitudes, one-third reported neutral attitudes, and 7.1% reported negative attitudes. This figure is lower than global surveys, where nearly 79% of respondents held positive views [15]. Regional studies have similarly reported higher positivity rates, with approximately 75% in Egypt [17] and favorable trends in Saudi Arabia [16]. Western experiences also show strong acceptance: US oncologists’ satisfaction rose to 81.5% post-COVID-19 [18]. Compared with these contexts, UAE physicians appear more cautious, though a clear majority remain supportive.

The results indicated that two-thirds of UAE physicians demonstrated good practice. Comparable findings have been reported internationally: US rural doctors adopted telemedicine at high rates [19], while 78% of Indonesian clinicians reported satisfaction with its use [18]. Regional studies also confirm rising adoption; for example, half of Egyptian physicians had used telemedicine, with most others willing to do so [17]. Notably, our study revealed significant geographic variation, reflecting the dynamic stage of telemedicine adoption and the potential for further harmonization across regions.

Multivariate models showed the effect of specialty, location, and facility type. Obstetrics and gynecology specialists had decreased odds of high knowledge compared to surgeons, while physicians in medicine and OB/GYN had increased odds of positive attitudes. Dubai-based physicians were more likely to report good practice, though they surprisingly had lower adjusted odds of high knowledge when compared with Sharjah physicians. Facility types also mattered: private-sector physicians had twice the odds of high knowledge as public-sector counterparts.

Interestingly, Dubai-based physicians demonstrated higher odds of good telemedicine practice despite lower adjusted odds of high knowledge. This apparent discrepancy may reflect the influence of system-level factors, such as greater availability of digital infrastructure, institutional support, and integration of telemedicine services, which may facilitate practice independent of formal knowledge levels. It is also possible that knowledge, as assessed in this study, reflects theoretical understanding rather than practical familiarity. Alternatively, this finding may be influenced by residual confounding or differences in sample distribution across emirates and should therefore be interpreted with caution. Further studies are needed to explore this relationship in more depth.

These variations may partially reflect structural characteristics of the UAE healthcare system. Healthcare delivery in the UAE operates through a dual public-private model, where private institutions often adopt new digital health technologies more rapidly due to greater operational flexibility and resource availability. This may partly explain the higher knowledge levels observed among physicians working in private facilities. In addition, variations across emirates may be influenced by differences in local health authority initiatives, digital infrastructure, and institutional implementation of telemedicine platforms. For example, emirate-level digital health initiatives and telemedicine services introduced by authorities such as the DHA may contribute to differing levels of physician exposure, training opportunities, and integration of telemedicine into routine clinical practice.

Differences across specialties may also reflect the varying clinical applicability of telemedicine. Specialties such as family medicine and internal medicine may be more adaptable to remote consultation models due to their reliance on history-based assessments, follow-up care, and chronic disease management, whereas procedural specialties may face greater limitations in delivering care remotely.

Compared with Western contexts, knowledge and acceptance of telemedicine often exceed 70-80% [14,15,18]. UAE physicians demonstrate more moderate levels. Within the MENA region, findings vary, but Egypt and Saudi Arabia generally report higher positivity than observed here [16,17]. The UAE, therefore, represents a middle ground: adoption is substantial but not yet universal, with variability linked to demographics and health system factors. Addressing specialty-specific gaps, supporting public-sector staff, and reducing geographic disparities may help align UAE performance with global benchmarks.

A crucial factor in telemedicine usability is patients’ familiarity with the system. In a previous cross-sectional survey assessing telemedicine usability among patients, 66.9% (678 of 1,013 participants) were well informed about telemedicine. Among those, 60.3% gained awareness of telemedicine through the internet and social media, while 50.7% cited hospitals and healthcare providers as their primary source. Despite this familiarity, actual usage remained low, with only 29.5% having used telemedicine services, primarily for consultations (31.2%) and prescription refills (29.7%) [20]. This gap between awareness and usage may suggest a lack of trust or confidence in the telemedicine system. Another study exploring digital healthcare in the UAE, which included both AI and telemedicine, identified key barriers to adoption such as data privacy concerns, high implementation costs, resistance from healthcare workers, and the need for proper training [21]. While telemedicine offers several advantages, including efficient communication, reduced time and cost, and enhanced access to primary care, patient preferences and perceptions continue to influence adoption. These factors should be addressed to fully integrate telemedicine into clinical practice [22].

This study offers several notable strengths that enhance its value and relevance. By targeting physicians, the research provides critical insights into the perspectives of healthcare providers who are central to the adoption and implementation of telemedicine in clinical practice, a dimension often underrepresented in the literature. Importantly, the study compared healthcare facilities across all seven emirates of the UAE, encompassing both government and private institutions, as well as physicians from a range of specialties and healthcare settings. This diversity provided a broader understanding of telemedicine across different contexts. Furthermore, the structured quantitative design allowed for recognition of patterns and determinants that influence telemedicine use. A nonprobability sampling strategy, combining convenience and snowball techniques, was employed to efficiently recruit participants. Conducted in the UAE, a country that has rapidly integrated innovative digital health solutions, the study offers insights that are not only locally significant but also relevant to global healthcare systems that strive to incorporate telemedicine into routine care. These findings have important implications for health system planning, particularly in standardizing telemedicine training across specialties and healthcare sectors. Strengthening institutional policies and regulatory frameworks may help reduce variability in adoption and ensure more consistent implementation across regions.

Despite its valuable contribution, this study has several limitations that should be acknowledged. The cross-sectional design captures physician perspectives at a single point in time, which limits the ability to infer causal relationships or determine temporal directionality between physician characteristics and telemedicine-related KAP. Future longitudinal or prospective studies would be valuable in assessing how these factors evolve over time and in identifying causal determinants of telemedicine adoption among physicians. Reliance on self-reported data introduces the possibility of response bias, including social desirability bias, as participants may overestimate positive experiences or underreport challenges. Additionally, the use of a nonprobability sampling strategy, including convenience and snowball sampling, may have introduced selection bias. Physicians with greater familiarity or interest in telemedicine may have been more likely to participate, potentially leading to an overrepresentation of individuals with favorable attitudes or higher engagement with telemedicine. Finally, although the study included physicians from multiple specialties and healthcare settings across several emirates, the relatively modest sample size may not fully represent the broader physician population in the UAE. As a result, the generalizability of the findings should be interpreted with caution. As telemedicine continues to shape the future of healthcare, further studies with larger, more diverse populations and a systematic approach to evaluating various technologies are essential [23].

## Conclusions

This study provides insights into physicians’ KAP regarding telemedicine across the UAE. The findings demonstrate generally favorable acceptance of telemedicine, alongside variability across specialties and healthcare settings. These results highlight the need for targeted interventions, including structured training programs and clear regulatory frameworks, to support consistent and effective implementation. Strengthening these areas may facilitate more equitable and sustainable integration of telemedicine into routine clinical practice. Further research is warranted to explore contextual and system-level factors influencing telemedicine adoption across different healthcare settings.

## Figures and Tables

**Figure 1:**
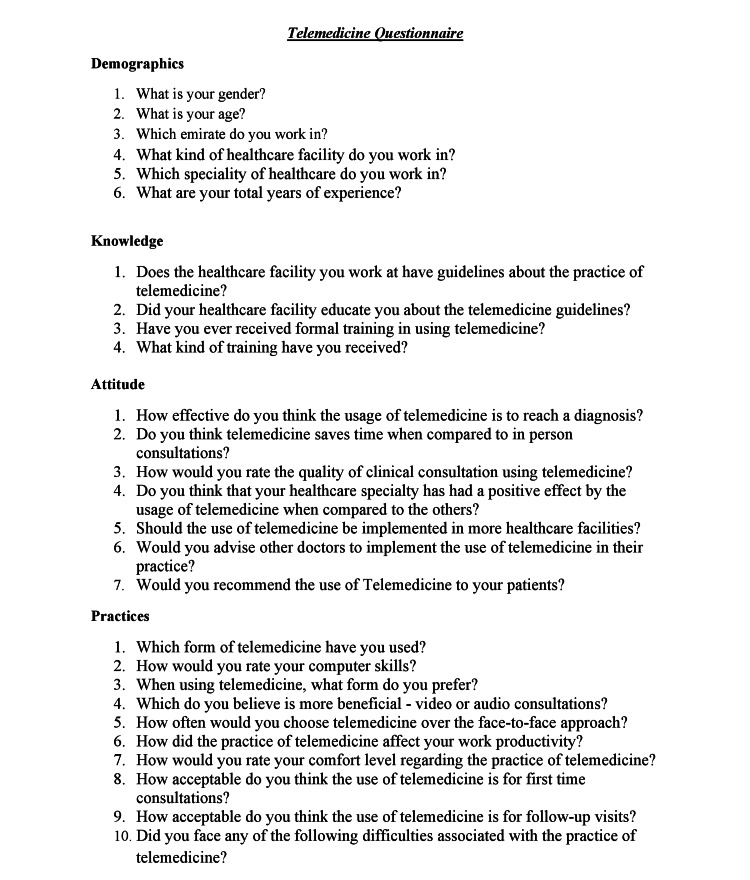
Telemedicine questionnaire

## Appendices

Appendix A: Questionnaire 
